# Measuring organisational readiness for patient engagement (MORE): an international online Delphi consensus study

**DOI:** 10.1186/s12913-015-0717-3

**Published:** 2015-02-14

**Authors:** Linda JM Oostendorp, Marie-Anne Durand, Amy Lloyd, Glyn Elwyn

**Affiliations:** Department of Psychology, Centre for Lifespan and Chronic Illness Research, University of Hertfordshire, Hatfield, UK; The Dartmouth Institute for Health Policy and Clinical Practice, HB 7256, Dartmouth College, Hanover, NH 03755 USA; Institute of Primary Care and Public Health, School of Medicine, Cardiff University, Heath Park, Cardiff, UK; The Dartmouth Center for Health Care Delivery Science, Dartmouth College, Hanover, USA

**Keywords:** Patient engagement, Implementation, Organisational readiness, Willingness, Ability, Scale development, Delphi consensus procedure

## Abstract

**Background:**

Widespread implementation of patient engagement by organisations and clinical teams is not a reality yet. The aim of this study is to develop a measure of organisational readiness for patient engagement designed to monitor and facilitate a healthcare organisation’s willingness and ability to effectively implement patient engagement in healthcare.

**Methods:**

The development of the MORE (Measuring Organisational Readiness for patient Engagement) scale was guided by Weiner’s theory of organisational readiness for change. Weiner postulates that an organisation’s readiness is determined by both the willingness and ability to implement the change (i.e. in this context: patient engagement). A first version of the scale was developed based on a literature search and evaluation of pre-existing tools. We invited multi-disciplinary stakeholders to participate in a two-round online Delphi survey. Respondents were asked to rate the importance of each proposed item, and to comment on the proposed domains and items. Second round participants received feedback from the first round and were asked to re-rate the importance of the revised, new and unchanged items, and to provide comments.

**Results:**

The first version of the scale contained 51 items divided into three domains: (1) Respondents’ characteristics; (2) the organisation’s willingness to implement patient engagement; and (3) the organisation’s ability to implement patient engagement. 131 respondents from 16 countries (health care managers, policy makers, clinicians, patients and patient representatives, researchers, and other stakeholders) completed the first survey, and 72 of them also completed the second survey. During the Delphi process, 34 items were reworded, 8 new items were added, 5 items were removed, and 18 were combined. The scale’s instructions were revised. The final version of MORE totalled 38 items; 5 on stakeholders, 13 on an organisation’s willingness to implement, and 20 on an organisation’s ability to implement patient engagement in healthcare.

**Conclusions:**

The Delphi technique was successfully used to refine the scale’s instructions, domains and items, using input from a broad range of international stakeholders, hoping that MORE can be applied in a variety of healthcare contexts worldwide. Further assessment is needed to determine the psychometric properties of the scale.

## Background

Promoting patient engagement in routine clinical care is considered one of the pillars of modern medicine [1,2]. In the UK, patient engagement has received renewed policy support in the form of the National Commissioning framework [3], and the Commissioning for Quality and Innovation (CQUIN) framework, where both enable commissioners to reward implementation initiatives. Further, several national programmes have emerged to stimulate shared decision-making (SDM) and self-management, which are important aspects of patient engagement [2]. This includes the National Voices and MAGIC websites, which provide a broad evidence base on how to best support self-management and SDM [4], and projects exploring and promoting the implementation of SDM [5,6]. Still, despite increasing policy commitments, routine adoption remains difficult [7,8]. Various definitions of patient engagement have been introduced [9]. Angela Coulter, in her book ‘Engaging patients in healthcare’ [2], defines patient engagement as “working together to promote and support active patient and public involvement in health and healthcare and to strengthen their influence on healthcare decisions, at both the individual and collective level”. Sometimes, the term ‘patient engagement’ is used interchangeably with terms such as ‘patient involvement’ or ‘patient activation’. However, these terms do not reflect all aspects of patient engagement, including the reciprocity between patients and health professionals or healthcare organisations [2,10].

Patient engagement in healthcare at the individual level can occur in direct care interactions, for example through SDM and at the collective level in the form of organisational design and governance [10]. Most work to date has focused on engaging patients at the individual level, e.g. barriers to implementing SDM at the health professional’s and patient’s levels have been explored [11,12]. When promoting engagement in organisational activities such as the commissioning, planning, design, delivery, evaluation, and reconfiguration of health services, patients and members of the public will need to be enabled to be engaged [13]. So far, studies have focused on barriers to engagement among health professionals and patients, but the impact of organisational barriers and facilitators on the spread and adoption of patient engagement in healthcare has not been closely investigated.

A complex whole system change such as patient engagement in healthcare would require its adoption by the organisation as a whole [14]. It is widely recognised that organisational changes are challenging, and often unsuccessful [15]. Some have suggested that half of all failures to implement organisational change can be ascribed to insufficient organisational readiness for change [16]. Organisational readiness for change can be defined as a state of being willing and able to take action [17]. A timely assessment of organisational readiness can facilitate effective implementation by enabling a tailored implementation strategy. Despite extensive searches, to the best of our knowledge, no tools to assess organisational readiness for patient engagement in healthcare exist. A systematic review of the literature on the conceptualisation and measurement of organisational readiness for change by Weiner and colleagues yielded 43 instruments [18]. Many of these scales were not specific to the healthcare context, and data on reliability or validity was often lacking. Recently, Weiner and colleagues published a measure of organisational readiness for change in healthcare settings. However the items in this generic scale were not specifically focussed on achieving patient engagement [19].

Given this gap, we initiated work to develop such a measure. This study addressed the following research question: ‘Which domains and items should be included in a scale to monitor and facilitate a healthcare organisation’s willingness and ability to effectively implement patient engagement in healthcare?’. As we were striving to develop a scale that would fit in a variety of healthcare contexts, the development of this scale included the consultation of a broad range of international stakeholders in patient engagement.

## Methods

### Study design

A Delphi study was performed to seek consensus on the nature, number, and wording of the items to include in the scale. The Delphi approach consists of an iterative process that aims to combine the perspectives of a panel of experts into a group consensus [20]. It was decided to conduct two Delphi rounds, in anticipation of competing priorities and time constraints of relevant stakeholders in the field of patient engagement [21]. In two rounds of online questionnaires, a panel of stakeholders was consulted to provide feedback about the evolving set of items. The anonymous responses from participants in round 1 were fed back to them in round 2.

### Participants

To maximise the generalisability of the results to a variety of healthcare contexts worldwide, we planned to include healthcare managers, policy makers, medical clinicians, nurse clinicians, other health professionals, administrative staff, patients and patient representatives, and researchers from various countries. Members of the research team nominated multi-disciplinary stakeholders on the basis of their interest and expertise in patient engagement. However, these experts all work in the field of patient engagement and most were acquainted with the researchers. In an effort to include a wider range of perspectives, additional open invitations were circulated through national and international mailing lists including the Evidence Based Medicine mailing list, the Shared Decision Making Network, the Shared Decision Making listserv, and the NHS CHAIN network. The invitations provided a brief outline of the study, an enclosed participant information sheet, and a link to the online survey. Consent was inferred by participants’ completion of the survey. Ethical approval was granted on 3 September 2013, by the University of Hertfordshire’s Ethics Committee for Studies Involving the Use of Human Participants (LMS/SF/UH/00024).

### Theoretical framework

A literature search on appropriate theories to underpin the development of the proposed new scale identified Weiner’s theory of organisational readiness for change in healthcare settings [17]. This theory was developed based on a comprehensive review of the literature on how organisational readiness has been defined and measured [18]. Weiner postulates that organisational readiness for change can be viewed as a psychological state that is shared by the members of the organisation, and in which they feel committed to implementing an organisational change (change commitment or ‘willingness to change’), and are confident in their collective abilities to do so (change efficacy or ‘ability to change’). Further, Weiner defined three key determinants of an organisation’s ability to change: task demands (‘do we know what it will take to implement this change effectively?’), resource availability (‘do we have the resources to implement this change effectively’), and situational factors (‘can we implement this change effectively given the situation we currently face?’).

### Questionnaire development

A literature search was conducted to identify any publications describing the development or validation of relevant scales. Four instruments that were broadly related to our planned approach were identified. This included a hospital self-assessment inventory for patient- and family-centred care [22], an organisational self-assessment tool for patient-centred care [23], a checklist for organisational readiness for improving patient care experience [24], and an organisational communication climate assessment toolkit [25].

We designed the domains of the ‘Measuring Organisational Readiness for patient Engagement’ (MORE) scale according to Weiner’s theory and developed items that were aligned with the theory’s key constructs, and in part derived from existing scales [22-25]. This was an iterative process involving regular discussions in the research team.

### Round 1 survey

The round one survey included a brief introduction to the aim of the study and a summary of the development process and theoretical underpinnings. Participants were asked to rate each of the proposed items on a four-point Likert scale. For the items about the stakeholders targeted by the scale, participants were asked to rate whether each of the proposed stakeholders should be involved (1 = avoid; 4 = definitely involve). For all other items, participants were asked to rate the importance of the items (1 = not important; 4 = very important). Furthermore, for each subset of items and at the end of the questionnaire, participants were given the opportunity to provide rewording suggestions, additional comments, or questions. Email addresses were collected for inclusion in round 2. Participants were also asked to complete basic demographic questions. The survey was open for three weeks and two email reminders were sent to the stakeholders on the contact list.

### Round 2 survey

Round 1 participants were invited to complete a second survey, in which feedback was provided about the items’ ratings of importance (percentage of participants who thought an item was important or very important) and about the changes made based on the qualitative feedback. Participants were invited to re-rate the importance of the items and to provide additional rewording suggestions, comments or questions. The same demographic items were included. The survey was open for three weeks and two email reminders were sent.

### Analysis

Following round 1, the research team discussed the qualitative feedback received, including rewording suggestions per item, suggestions to add new items and more general comments or questions. Items were revised if at least two participants suggested it, or if the researchers agreed that the item would benefit from rewording or merging. For example, for item a in domain 1 ‘Informing patients about their condition or potential health issues’, two participants suggested changing ‘informing’ to ‘discussing’ and one participant suggested using the phrase ‘engaging patients in..’. For round 2, the item was reworded accordingly to ‘Engaging patients in discussing their condition or potential health issues’.

Following the two survey rounds, a decision on whether to retain items in the scale was made based on the ratings of importance in round 2. The ratings were summarised using percentages and the views of all participants were given equal weight. If at least 70% of participants rated the importance of the item in the lower two categories (not important or somewhat important), or in the higher two categories (important or very important), it was determined that consensus was achieved and the item would be removed or retained, respectively. A 70% threshold was considered appropriate, considering that the MORE scale will undergo further testing, and is consistent with related research using a Delphi approach [26,27]. If no consensus was achieved, the research team decided whether or not to retain an item in the scale, basing this decision on qualitative feedback from the participants where possible. For example, no consensus was achieved on whether to retain gender, age and length of employment in domain 1 of the scale. The research team decided to retain these items to examine the representativeness of the stakeholders completing the tool, supported by qualitative feedback from multiple participants emphasising the importance of capturing as many different perspectives as possible.

## Results

### Participants

The first Delphi round took place between 15 November 2013 and 6 December 2013. Of the 69 participants nominated in the contact list, 30 completed the questionnaire (43%). In addition, 101 questionnaires were completed by participants recruited through the open invitations, thus totalling 131 participants (see Table 1 for participants’ characteristics). Sixteen countries were represented in this study: UK (*n* = 49), US (*n* = 33), the Netherlands (*n* = 13), Germany (*n* = 11), Canada (*n* = 6), Austria (*n* = 2), India (*n* = 2), Ireland (*n* = 2), Israel (*n* = 2), Italy (*n* = 2), New Zealand (*n* = 2), Spain (*n* = 2), Australia (*n* = 1), Denmark (*n* = 1), Romania (*n* = 1), Switzerland (*n* = 1), and Europe (country unspecified) (*n* = 1). The second round took place between 11 and 28 February 2014 and was completed by 72 of the 131 invited round 1 participants (55%) (see Table 1).

**Table 1:** **Participants’ characteristics**

	**Round 1**					**Round 2**				
**Role**	**n (%)**	**Time in role**	**Gender**	**Age**	**Mother-tongue**	**n (%)**	**Time in role**	**Gender**	**Age**	**Mother-tongue**
Healthcare manager	n = 13 (10%)	6 mo-2 yrs (3) 2–5 yrs (4) >5 yrs (6)	female (7) male (6)	36-50 yrs (7) >50 yrs (6)	English (12) Other (1)	n = 4 (6%)	2-5 yrs (2) >5 yrs (2)	female (2) male (2)	36-50 yrs (2) >50 yrs (2)	English (4)
Policy maker	n = 4 (3%)	6 mo-2 yrs (2) 2–5 yrs (1) >5 yrs (1)	female (4)	36-50 yrs (2) >50 yrs (2)	English (2) Other (2)	n = 5 (7%)	6 mo-2 yrs (2) 2–5 yrs (1) >5 yrs (2)	female (4) male (1)	36-50 yrs (3) >50 yrs (2)	English (4) Other (1)
Clinicians/other health professionals	n = 36 (27%)	<6 mo (1) 6 mo-2 yrs (2) 2–5 yrs (5) >5 years (28)	female (17) male (19)	25-35 yrs (3) 36–50 yrs (14) >50 yrs (19)	English (25) Other (11)	n = 26 (36%)	6 mo-2 yrs (1) 2–5 yrs (1) >5 years (24)	female (14) male (12)	25-35 yrs (1) 36–50 yrs (13) >50 yrs (12)	English (17) Other (9)
Administration: receptionist	n = 0 (0%)	-	-	-	-	-	-	-	-	-
Administration: other	n = 0 (0%)	-	-	-	-	-	-	-	-	-
Patients and patient representatives	n = 23 (18%)*	<6 mo (1) 6 mo-2 yrs (4) 2–5 yrs (5) >5 yrs (13)	female (18) male (5)	25-35 yrs (1) 36–50 yrs (4) >50 yrs (18)	English (19) Other (4)	n = 12 (17%)	6 mo-2 yrs (4) 2–5 yrs (2) >5 yrs (6)	female (10) male (2)	25-35 yrs (1) 36–50 yrs (2) >50 yrs (9)	English (9) Other (3)
Academic	n = 52 (40%)	<6 mo (2) 6 mo-2 yrs (4) 2–5 yrs (5) >5 yrs (41)	female (34) male (18)	25-35 yrs (12) 36–50 yrs (17) >50 yrs (23)	English (31) Other (21)	n = 24 (33%)	2-5 yrs (3) >5 yrs (21)	female (17) male (7)	25-35 yrs (7) 36–50 yrs (8) >50 yrs (9)	English (11) Other (13)
Other	n = 3 (2%)	2-5 yrs (2) >5 yrs (1)	female (1) male (2)	36-50 yrs (1) >50 yrs (2)	English (2) Other (1)	n = 1 (1%)	>5 yrs (1)	female (1)	>50 yrs (1)	Other (1)

*Including 22 patient representatives and 1 patient.

### Development of the first draft of the scale

A total of 51 items were developed, divided into three domains. The first domain contained items related to the background of the stakeholders involved in completing the scale, to ensure that these stakeholders were representative of the entire workforce (see Table 2). Following Weiner’s theory, the second domain was designed to assess the organisation’s willingness to implement patient engagement and included 14 items reflecting the different aspects of patient engagement (at the individual and collective levels) (e.g. ‘Informing patients about their condition or potential health issues’). These items were partly derived from existing scales [22-25] and complemented with newly developed items to reflect all aspects of patient engagement. The third domain was designed to assess the organisation’s ability to implement patient engagement, in terms of tasks (9 items, e.g. ‘Developing a shared organisational vision for patient engagement’), resources (10 items; e.g. ‘Expertise in patient engagement’), and situational factors (7 items; e.g. ‘Alignment of patient engagement with organisational priorities’). Again, items in these domains were partly derived from existing scales [22-25] and complemented with newly developed items. In addition, the online questionnaire contained six questions about the demographics of the Delphi survey participants. It was developed using Bristol Online Survey [28]. To support face and content validity, the survey was pilot tested among colleagues.

**Table 2:** **Participants’ ratings of the proposed items**

**DOMAIN 1: Stakeholders**				
**Type of stakeholders**				
	**Round 1**		**Round 2**	
**Item**	**Initial wording of the item**	**% of participants who thought these stakeholders should preferably or definitely be involved in completing the scale**	**Changes made in round 2**	**% of participants who thought these stakeholders should preferably or definitely be involved in completing the scale**
a.	Senior Managers	127 (97%)	*reworded:* Executives/Board of Directors	70 (97%)
b.	Junior Managers	124 (95%)	*reworded:* Managers	71 (99%)
c.	Medical Clinicians	129 (99%)	*unchanged*	72 (100%)
d.	Nurse Clinicians	130 (99%)	*unchanged*	72 (100%)
e.	Other Health professionals*	126 (96%)	*unchanged*	72 (100%)
f.	Receptionists	99 (76%)	*unchanged*	62 (86%)
g.	Other staff in administration	87 (66%)	*unchanged*	57 (79%)
h.	Other	76 (58%)	*unchanged*	47 (65%)
**Stakeholders’ characteristics**				
	**Round 1**		**Round 2**	
**Item**	**Initial wording of the item**	**% of participants who thought the item was important or very important**	**Changes made in round 2**	**% of participants who thought the item was important or very important**
a.	Gender	50 (38%)	*unchanged*	34 (47%)
b.	Age	66 (50%)	*unchanged*	38 (53%)
c.	Length of employment	80 (61%)	*unchanged*	43 (60%)
d.	-	-	*new:* Role in the organisation	61 (85%)
e.	-	-	*new:* Discipline (e.g. cardiology)	49 (68%)
f.	-	-	*new:* Ethnicity	26 (36%)
**DOMAIN 2: The organisation’s willingness to implement patient engagement**				
	**Round 1**		**Round 2**	
**Item**	**Initial wording of the item**	**% of participants who thought the item was important or very important**	**Changes made in round 2**	**% of participants who thought the item was important or very important**
a.	Informing patients about their condition or potential health issues	126 (96%)	*reworded:* Engaging patients in discussing their condition or potential health issues	72 (100%)
b.	Informing patients about all available options, and the potential benefits and risks of each option	128 (98%)	*reworded*: Discussing all relevant health care options with patients (including doing nothing), and the potential benefits and risks of each option	71 (99%)
c.	Actively checking patients’ understanding	128 (98%)	*unchanged*	72 (100%)
d.	Encouraging patients to ask questions and voice concerns	130 (99%)	*unchanged*	72 (100%)
e.	Engaging patients in collaborative decision-making	129 (99%)	*reworded:* Encouraging patients to make health care decisions in partnership with the health care team	71 (99%)
f.	Engaging patients in positive health behaviours	110 (84%)	*unchanged*	61 (85%)
g.	Eliciting patients’ preferences	128 (98%)	*combined with item h. and reworded:* Asking patients about their health-related preferences and acting upon them	71 (99%)
h.	Taking patient preferences into account	128 (98%)	*combined with item g.*	-
i.	Providing patients with written information	109 (83%)	*combined with item j. and reworded:* Supporting patients with additional health information resources (e.g. access to patient groups and decision support resources)	67 (93%)
j.	Providing patients with decision support tools	111 (85%)	*combined with item i.*	-
k.	Ensuring effective oral and written communication with diverse patients	123 (94%)	*reworded:* Communicating with patients in a format that all patients can understand	71 (99%)
l.	Giving patients access to their medical information	115 (88%)	*reworded:* Supporting patients to access their medical information in a format they can understand	71 (99%)
m.	Asking patients for feedback about their care experiences	124 (95%)	*reworded:* Asking patients for feedback about their care experiences and acting upon it	70 (97%)
n.	Engaging patients as advisors in the organisation	114 (87%)	*reworded:* Engaging patients as partners in the organisation in all areas of health care services (e.g. design, delivery, and evaluation)	61 (85%)
o.	-	-	*new:* Treating patients as partners, with respect and consideration for their individual needs, throughout their care journey, from entering the building to receiving reatments etc.	71 (99%)
p.	-	-	*new:* Developing care plans as a partnership between health professionals and patients with long term conditions (e.g. defining mutually agreed goals, actions, and timeframes)	71 (99%)
**DOMAIN 3: The organisation’s ability to implement patient engagement**				
**Tasks**				
	**Round 1**		**Round 2**	
**Item**	**Initial wording of the item**	**% of participants who thought the item was important or very important**	**Changes made in round 2**	**% of participants who thought the item was important or very important**
a.	Developing a shared organisational vision for patient engagement	126 (96%)	*reworded:* Developing a shared organisational vision for patient engagement among employees and patients	70 (97%)
b.	Getting ownership of a shared organisational vision for patient engagement	107 (82%)	*reworded:* Sharing the organisational vision for patient engagement with all employees	69 (96%)
c.	Sharing the organisational vision for patient engagement with all patients	114 (87%)	*reworded:* Sharing the organisational vision for patient engagement with all patients and the public (e.g. information in waiting areas)	66 (92%)
d.	Including patient engagement in policies, processes, position descriptions and training programs	119 (91%)	*reworded:* Including patient engagement in all areas of health care services (e.g. policies, processes, position descriptions and training programs)	68 (94%)
e.	Tailoring communication to individual patients’ needs	126 (96%)	*removed because this item is covered in domain 2*	-
f.	Tailoring consultations to individual patients’ needs	123 (94%)	*removed because this item is covered in domain 2*	-
g.	Supporting all employees in their efforts to promote patient engagement	128 (98%)	*reworded:* Supporting employees in their efforts to promote patient engagement (e.g. asking what they need and addressing these needs, reminders)	71 (99%)
h.	Monitoring patient engagement in the organisation	124 (95%)	*reworded:* Monitoring patient engagement in the organisation and giving feedback to employees	72 (100%)
i.	Solving problems that arise during the implementation of patient engagement	128 (98%)	*unchanged*	72 (100%)
**Resources**				
	**Round 1**		**Round 2**	
**Item**	**Initial wording of the item**	**% of participants who thought the item was important or very important**	**Changes made in round 2**	**% of participants who thought the item was important or very important**
a.	Expertise in patient engagement	118 (90%)	*reworded:* Access to expertise in patient engagement	71 (99%)
b.	Time for initial implementation of patient engagement	123 (94%)	*reworded:* Time for initial implementation of patient engagement (e.g. time to inform employees about patient engagement processes)	70 (97%)
c.	Time for monitoring implementation of patient engagement	125 (95%)	*reworded:* Time for monitoring implementation of patient engagement (e.g. time for employees to provide feedback)	69 (96%)
d.	Time to make patient engagement happen	125 (95%)	*reworded:* Time to make patient engagement happen (e.g. revising targets and objectives, longer consultations)	72 (100%)
e.	Communication skills training	121 (92%)	*combined with item f. and reworded:* Training health professionals in patient engagement (e.g. communication and shared decision-making skills)	72 (100%)
f.	Training in patient engagement and shared decision-making skills	128 (98%)	*combined with item e.*	-
g.	Patient education materials in a language the patient can understand	123 (94%)	*combined with item h. and i. and reworded:* Resources to provide health-related information and support to patients (e.g. access to interpreters, answering questions, helping patients to make decisions)	72 (100%)
h.	Decision support resources	113 (86%)	*combined with item g. and i.*	-
i.	Trained medical interpreters and care coordinators	105 (80%)	*combined with item g. and h.*	-
j.	Systems and processes that can identify and adapt to diverse patients’ needs	116 (89%)	*reworded:* Systems and processes that can adapt to diverse patients’ needs (e.g. scheduling of appointments)	68 (94%)
k.	-	-	*new:* Access to patient representatives	57 (79%)
l.	-	-	*new:* Resources to support patients in becoming partners (e.g. recruitment of representatives, training, coaching, money to pay patients for participation)	59 (82%)
m.	-	-	*new:* Tools to evaluate the implementation of patient engagement	68 (94%)
**Situational factors**				
	**Round 1**		**Round 2**	
**Item**	**Initial wording of the item**	**% of participants who thought the item was important or very important**	**Changes made in round 2**	**% of participants who thought the item was important or very important**
a.	Alignment of patient engagement with organisational priorities	123 (94%)	*unchanged*	68 (94%)
b.	Timing of the implementation of patient engagement	105 (80%)	*removed because this is already covered by item a.*	-
c.	Employee attitudes, beliefs, and experiences regarding patient engagement	126 (96%)	*unchanged*	70 (97%)
d.	Positive and consistent communication about patient engagement	126 (96%)	*reworded:* Frequent and consistent communication about patient engagement	69 (96%)
e.	Employee involvement in planning the implementation of patient engagement	127 (97%)	*reworded:* Employee involvement in planning, implementation, and monitoring of patient engagement	71 (99%)
f.	Patient involvement in planning the implementation of patient engagement	117 (89%)	*reworded:* Patient involvement in planning, implementation, and monitoring of patient engagement	70 (97%)
g.	Performance measures include patient engagement	120 (92%)	*unchanged*	66 (92%)

*(e.g. Clinical Psychologists or Allied Health professionals).

### Survey results

Participants’ ratings of importance are shown in Table 2. In round 1, 222 rewording suggestions, 238 other suggestions and 23 general comments were provided. In round 2, 91 rewording suggestions, 42 other suggestions, and 8 general comments were made. Below, the results of the survey are presented per domain of the scale, with each round described separately.

### Domain 1: stakeholders involved in completing the scale

#### Round 1

Between 95 and 99% of all participants agreed that senior and junior managers, clinicians and other health professionals should be involved in completing the scale. The majority of participants also agreed to include the following staff categories: receptionists, other staff in administration, and other staff. Several participants commented that executives or members of the organisation’s Board should also be involved in completing the scale. The items ‘Senior Managers’ and ‘Junior Managers’ were reworded to ‘Executives/Board of Directors’ and ‘Managers’. Other feedback related to involving patients in completing the scale. However, given many of the items do not apply to individual patients, a recommendation to involve patient representatives will therefore be included in the instructions. In the future, we will consider developing a separate version of the scale specifically intended for patients. Assessing the gender, age, and length of employment of stakeholders completing the scale was believed to be important or very important by 38%, 50%, and 61% of the survey participants. Based on the qualitative feedback, three new items were added in round 2: (1) ‘role in the organisation’, (2) ‘discipline (e.g. cardiology)’ and (3) ‘ethnicity’.

#### Round 2

Participants’ ratings of staff categories were similar to round 1. The items ‘Other staff in Administration’ and ‘Other’ were merged into: ‘Other stakeholders’. Participants also suggested a number of additional stakeholders (e.g. HR staff). These will be included in the scale’s instructions as an example of ‘Other stakeholders’. The ratings of importance for gender, age and length of employment were similar to round 1 and no consensus was reached (47-60%). The research team decided to retain these items to examine the representativeness of the stakeholders completing the scale. For the three new items (‘role in the organisation’ (85%), followed by ‘discipline’ (68%) and ‘ethnicity’ (36%)), consensus was reached that ‘role in the organisation’ would be retained, with an open question in which stakeholders can also report their discipline. The ‘ethnicity item’ was removed from the scale.

### Domain 2: The organisation’s willingness to implement patient engagement

#### Round 1

Most participants (83-99%) considered that it would be ‘important’ or ‘very important’ to include all 14 items when assessing an organisation’s willingness to implement patient engagement. Six items were reworded, which essentially consisted of representing the patient’s voice more strongly and using plainer language. Furthermore, the two items about eliciting patients’ preferences and taking patient preferences into account were combined and reworded into ‘Asking patients about their health-related preferences and acting upon them’. The two items about provision of written information and decision support tools were perceived to be overlapping and merged into ‘Supporting patients with additional health information resources (e.g. access to patient groups and decision support resources)’. Two new items were added in order to emphasise the importance of including self-management as a key aspect of patient engagement, as well as the role of all members of the organisation in treating patients as partners. Further, some participants suggested broadening the definition of ‘patients’ by emphasising the role of families and carers. Others believed that the term ‘patient’ would not apply to healthy people using health services (e.g. maternity services or screening). An explanation of this term will be provided in the instructions of each domain of the scale.

#### Round 2

Similarly to round 1, all 14 items were rated very highly (85-100%). For the nine combined and/or reworded items, ratings were similar or higher than round 1 ratings. The newly added items were rated very highly (99%). Based on participants’ suggestions, three of the items were reworded to emphasise that communication should be tailored to individual patients. The newly added item about treating patients as partners, with respect and consideration for their individual needs, was removed based on the comment that this would be difficult to operationalise and measure.

### Domain 3: The organisation’s ability to implement patient engagement

#### Tasks

##### Round 1

All ten items in this domain were rated very highly (83-99%). Six items were reworded, three of which were clarified by adding examples. Two items about tailoring communication and consultations to individual patients’ needs were removed because of perceived duplication.

##### Round 2

Ratings of importance were consistently high or higher than in round 1 (92-100%). Following participants’ comments, the two items about sharing the organisational vision for patient engagement were merged into: ‘Sharing the organisational vision for patient engagement with employees, patients, and the public (e.g. information on intranet, information in waiting areas)’. In line with this change, the item about monitoring patient engagement and giving feedback to employees was reworded accordingly: ‘Monitoring patient engagement in the organisation and giving feedback to employees, patients, and the public’. Further, the item about solving problems was broadened to encompass both individual issues and organisational improvements.

#### Resources

##### Round 1

Participants gave all ten items a high rating of importance (80-98%). Two items about training were combined into the following: ‘Training health professionals in patient engagement (e.g. communication and shared decision-making skills)’. We also combined three items about patient education materials, decision support resources, and trained medical interpreters and care coordinators: ‘Resources to provide health-related information and support to patients (e.g. access to interpreters, answering questions, helping patients to make decisions). In addition, we reworded five items and added examples. Three new items about access to patient representatives, having resources to support patients in becoming partners, and tools to evaluate the implementation of patient engagement were added.

##### Round 2

Increased ratings of importance were seen for all seven combined or reworded items (94-100%). In addition, high ratings were obtained for the three new items (79-94%). The items addressing the impact of time on patient engagement were reworded to account for the fact that not only time, but other resources too, would be required. Some participants felt that the item on adaptable systems and processes was unclear. This item was merged with the item about resources to provide health-related information and support to patients: ‘Resources to provide health-related information and support to patients to meet their diverse needs (e.g. access to interpreters, answering questions, scheduling appointments)’. Other participants found the item about access to patient representatives confusing. It was merged with the resources to support patients in becoming partners item into ‘Resources to support patients in becoming partners (e.g. access to patient representatives, recruitment of representatives, training, coaching, money to pay patients for participation)’.

#### Situational factors

##### Round 1

Ratings of importance ranged from 80 to 97%. The ‘timing of implementation item’ was removed because of perceived duplication. We reworded the item about ‘positive and consistent communication’ into ‘frequent and consistent communication’ to reflect the respondents’ belief that communication is not/should not be overly positive. Two items about employee and patient involvement in planning the implementation were reworded to reflect the necessary involvement of employees and patients in all aspects of patient engagement (planning, implementation, and evaluation).

##### Round 2

Compared to round 1, participants gave very similar or slightly higher ratings (92 to 97%). The item about organisational priorities was reworded to ‘Alignment of organisational priorities with patient engagement’ to better reflect that patients’ priorities should be the organisation’s priorities.

## Discussion

### Principal findings

This study describes the first steps in the development of a measure of organisational readiness for patient engagement in healthcare (MORE). Guided by Weiner’s theory, a first version of the scale was developed by the research team using items derived from related tools. One hundred and thirty one multidisciplinary stakeholders from 16 countries gave their feedback on the proposed instructions, domains, and items in an online Delphi consensus procedure. Based on stakeholders’ feedback, the number of proposed items was reduced from 51 to 38 by merging overlapping items and deleting items that would be difficult to operationalise and measure. Participants’ feedback was also helpful to word items in plainer language, to clarify items by adding examples, and improve the scale’s instructions.

### Strengths and limitations

This study is the first to report the development of a theory-based measure of organisational readiness for patient engagement. This work builds on the expertise and experience of the authors in developing measurement scales in the area of patient engagement in healthcare [29-32], and was complemented by that of the 131 international stakeholders. It is worth noting that 16 countries were represented, therefore promoting the potential applicability of the scale in various healthcare contexts. Although the broad selection of stakeholders is a strength, it may be considered a weakness due to the inability to establish the exact number and categories of stakeholders approached through open invitations. In addition, while our sampling approach targeted a variety of healthcare contexts, we cannot be sure that all healthcare contexts (e.g. primary care, nursing homes, etc.) were represented in our survey. Further, despite including the CHAIN network, no administrative staff completed the Delphi survey. This is considered a weakness as receptionists and other administrators of healthcare organisations have an important role to play in fostering patient engagement and are often the patient’s first port of call. Some groups, especially policy makers, seem to be underrepresented in the survey. While this could potentially be addressed by assigning more weight to the answers of this group, it would be difficult to decide on the weight that should be used, and there are no indications that policy makers rated the importance of the items differently compared to other participants. Another group that may have been underrepresented is patients. Only one of the participants identified himself as a patient. However, 22 participants identified themselves as patient representatives, describing a variety of roles including family members and patient advocates. Finally, the consistently high ratings of importance across all three domains suggest a ceiling effect. To improve the design of future Delphi studies, it has been suggested that ceiling effects can be reduced by replacing the 4-point scale with a 5-point scale, using three positive choices (moderately important, important, and very important) and two negative choices (not at all important, somewhat important) [33].

### Results in context

A sizeable proportion of commonly reported barriers to patient engagement in healthcare are admittedly influenced, and occasionally fostered, by organisational factors: Time, continuity of care, workflow, characteristics of the healthcare settings (e.g. noisy environments, lack of privacy), lack of skills to apply SDM and access to training, clinical coordination and inter-professional collaboration, overall attitudes towards patient engagement, competing priorities and targets (e.g. criteria for referral, reimbursement in favour of surgery) [34,35]. If patient engagement is to become a reality in routine care, it is critical to give organisations the means to identify and address those barriers through usable routine measurements.

As far as can be determined, MORE is the first measure to target organisational readiness for patient engagement in healthcare. However, other scales have focussed on related constructs. The Organisational Readiness for Implementing Change measure (ORIC) was based on Weiner’s theory of organisational readiness for change [19]. While ORIC’s initial psychometric assessment, combining a qualitative approach, lab studies and field testing show promises, convergent, predictive and discriminant validity still remain to be tested. Further, one may question the value of lab studies that did not include the target population with limited data in naturalistic settings. By contrast, Wynia’s validation study of an organisational communication climate assessment toolkit was conducted among its target users, across 13 geographically and ethnically diverse health care organisations [25]. The analysis suggested that domains and items were reliable and accurately predicted patients’ perceptions of the quality of organisational communication.

### Future research

Building on the above validation methodologies, and given our intention to develop a measure that can be applied in routine settings as well as being used for research purposes, we intend to prioritise naturalistic testing over simulated validation. We will use a combination of qualitative and quantitative methods. First, the items and domains of MORE will undergo further refinement using cognitive debriefing interviews with a wide range of international stakeholders. MORE was initially conceptualised as a measure that could be used across countries and healthcare settings (with minor adaptation and translation where necessary). We therefore intend to interview stakeholders from the US, UK and from at least one European country. In parallel, observation and field notes will be used to determine how the scale can fit in healthcare settings. Our ultimate goal is for MORE to become a routine measure, one that is not used solely for research purposes but to foster change and improvement and enable organisations to improve their practices, track progress and flag-up potential barriers to patient engagement. Second, we will assess the psychometric properties of the scale in a field study across geographically diverse healthcare organisations. We will assess intra-rater reliability, as well as convergent, discriminant and predictive validity. In accordance with Weiner’s theory, we will analyse within group agreement in order to determine whether ‘organisational readiness for patient engagement’ is indeed a psychological state shared by the members of an organisation.

## Conclusions

Promoting patient engagement in healthcare is critical to the quality and efficiency of healthcare delivery. This is often mediated, and arguably hindered, by organisational factors. It is therefore essential to facilitate an organisation’s transition to truly patient-centred services, where patients, their families and their carers are engaged throughout the care pathway, from booking an appointment to discussing treatment options with the medical team and being consulted on the design and delivery of services. The first prototype of MORE has been carefully developed using a solid theoretical and conceptual foundation, and iterative development process. The clarity, relevance and applicability of the items and domains have been significantly strengthened by the ratings and qualitative inputs of 131 multi-disciplinary stakeholders from 16 countries, including 18% of patients and patient representatives. Further assessment is needed to determine the psychometric properties of the scale.

## Abbreviations

CQUIN: Commissioning for Quality and Innovation
MORE: Measuring Organisational Readiness for patient engagement
ORIC: Organisational readiness for implementing change
SDM: Shared decision making
